# HMGA2 as a functional antagonist of PARP1 inhibitors in tumor cells

**DOI:** 10.1002/1878-0261.12390

**Published:** 2018-11-28

**Authors:** Sabine Hombach‐Klonisch, Forouh Kalantari, Manoj Reddy Medapati, Suchitra Natarajan, Sai Nivedita Krishnan, Aditya Kumar‐Kanojia, Thatchawan Thanasupawat, Farhana Begum, Fred Y. Xu, Grant M. Hatch, Marek Los, Thomas Klonisch

**Affiliations:** ^1^ Department of Human Anatomy and Cell Science Rady Faculty of Health Sciences Max Rady College of Medicine University of Manitoba Winnipeg Canada; ^2^ Department of Pharmacology & Therapeutics Rady Faculty of Health Sciences Max Rady College of Medicine University of Manitoba Winnipeg Canada; ^3^ Department of Biochemistry and Medical Genetics DREAM Children's Hospital Research Institute of Manitoba Rady Faculty of Health Sciences Max Rady College of Medicine University of Manitoba Winnipeg Canada; ^4^ Department of Małopolska Centre of Biotechnology Jagiellonian University Krakow Poland; ^5^ Department of Surgery Rady Faculty of Health Sciences Max Rady College of Medicine University of Manitoba Winnipeg Canada; ^6^ Department of Medical Microbiology & Infectious Diseases Rady Faculty of Health Sciences Max Rady College of Medicine University of Manitoba Winnipeg Canada; ^7^ Present address: Division of Radiation and Cancer Biology Department of Radiation Oncology Stanford University School of Medicine Stanford CA 94305 USA

**Keywords:** HMGA2, olaparib, PARP1, PARP1 trapping, PARylation

## Abstract

Poly(ADP‐ribose) polymerase 1 inhibitors alone or in combination with DNA damaging agents are promising clinical drugs in the treatment of cancer. However, there is a need to understand the molecular mechanisms of resistance to PARP1 inhibitors. Expression of HMGA2 in cancer is associated with poor prognosis for patients. Here, we investigated the novel relationship between HMGA2 and PARP1 in DNA damage‐induced PARP1 activity. We used human triple‐negative breast cancer and fibrosarcoma cell lines to demonstrate that HMGA2 colocalizes and interacts with PARP1. High cellular HMGA2 levels correlated with increased DNA damage‐induced PARP1 activity, which was dependent on functional DNA‐binding AT‐hook domains of HMGA2. HMGA2 inhibited PARP1 trapping to DNA and counteracted the cytotoxic effect of PARP inhibitors. Consequently, HMGA2 decreased caspase 3/7 induction and increased cell survival upon treatment with the alkylating methyl methanesulfonate alone or in combination with the PARP inhibitor AZD2281 (olaparib). HMGA2 increased mitochondrial oxygen consumption rate and spare respiratory capacity and increased NAMPT levels, suggesting metabolic support for enhanced PARP1 activity upon DNA damage. Our data showed that expression of HMGA2 in cancer cells reduces sensitivity to PARP inhibitors and suggests that targeting HMGA2 in combination with PARP inhibition may be a promising new therapeutic approach.

**Abbreviations**
ADP: adenosine diphosphate
DSB: double‐strand breaks
HMGA2: high‐mobility group A2
IF: immunofluorescence
IP: immunoprecipitation
KD: knock down
MEF: mouse embryonic fibroblasts
MMS: methyl methanesulfonate
NAD: nicotinamide adenine dinucleotide
NAMPT: nicotinamide phosphoribosyltransferase
PARG: Poly(ADP‐ribose) glycohydrolase
PARP1: Poly(ADP‐ribose) polymerase 1
PAR: Poly(ADP‐ribose)
PLA: proximity ligation assay
SSB: single‐strand breaks
TNBC: triple‐negative breast cancer
γH2AX: phosphorylated histone variant H2AX

## 1. Introduction

The role of poly(ADP‐ribose) polymerase 1 (PARP1) in sensing DNA damage and contributing to DNA damage repair is well recognized (De Vos *et al*., 2012; Kim *et al*., 2005). PARP1 serves as early sensor of nicked DNA and as a key factor in the acute DNA damage response (De Vos *et al*., 2012). PARP1 binds to DNA single‐strand breaks (SSBs) and double‐strand breaks (DSBs) with resulting activation of its catalytic activity (De Vos *et al*., 2012). PARP1 activation creates poly(ADP‐ribose) (PAR) using nicotinamide adenine dinucleotide (NAD) as a substrate and subsequently links linear and branched PAR chains onto PAR acceptor proteins, including PARP1 itself (Altmeyer *et al*., 2009) (Langelier and Pascal, 2013). This process is referred to as PARylation (poly‐(ADP‐ribose)ylation). PARylation is an important modulator of protein function and protein/protein/DNA interactions, and its emerging role as part of a protective cellular response to genomic stress is increasingly gaining attention (Jungmichel *et al*., 2013). PARP1 contributes to more than 80% of DNA damage‐induced PARylation, and 90% of PAR chains are linked to PARP1 itself (Altmeyer *et al*., 2009). PARP1 activation by DNA damage recruits proteins involved in DNA damage repair (Kim *et al*., 2005) and causes rapid and temporary relaxation of chromatin at the site of DNA damage which involves the temporary replacement of histone H1 (Strickfaden *et al*., 2016).

The involvement of PARP1 catalytic activity in single‐ and double‐strand DNA repair makes it an ideal therapeutic target in combination with DNA damaging agents or radiation (De Vos *et al*., 2012; Parsons *et al*., 2005). Therapeutically, the inhibition of PARP1 has been exploited in tumors with deficiencies in homologous recombination repair mechanisms and the FDA‐approved PARP1 inhibitor olaparib (Lynparza) is used in clinical trials in patients with advanced breast and ovarian tumors and germline BRCA1/2 mutations (Bryant *et al*., 2005; Farmer *et al*., 2005). The potential efficacy of PARP1 inhibitors in the treatment for other tumors with defects in DNA repair mechanisms or in combination with DNA damaging drugs warrants a much broader clinical use. However, resistance to PARP1 inhibitors as recently reviewed in Ref. (Montoni *et al*., 2013) indicates that several modulators of PARP activity exist which may contribute to insensitivity or resistance to PARP1 inhibitors. One factor linked to poor clinical outcome in many cancers is the nonhistone chromatin‐binding protein high‐mobility group protein A2 (HMGA2) (Fusco and Fedele, 2007). HMGA2 is usually undetectable in normal adult somatic cells (Gattas *et al*., 1999; Rogalla *et al*., 1996), but is expressed in embryonic tissues (Gattas *et al*., 1999; Rogalla *et al*., 1996) and embryonic stem (ES) cells (Droge and Davey, 2008), and re‐expressed in cancer (stem) cells (Fusco and Fedele, 2007; Yu *et al*., 2007). *HMGA2* expression is associated with cellular transformation (Berlingieri *et al*., 1995), directly correlates with the level of malignancy, and is linked to enhanced metastatic potential and poor clinical outcome in different cancers (Morishita *et al*., 2013; Sgarra *et al*., 2018). The expression of this oncofetal stem cell factor is regulated by the Lin28‐Let‐7 pathway (Hammond and Sharpless, 2008). Mutations to the 3’ untranslated region of the *HMGA2* gene can impair the binding of microRNA, including Let‐7, and increase HMGA2 protein expression. In breast tumors, increased Wnt/β‐catenin signaling was shown to upregulate HMGA2, promote EMT transformation, and increase tissue invasion of tumor cells (Wend *et al*., 2013).

HMGA2 utilizes its three lysine‐ and arginine‐rich AT‐hook domains to bind to AT‐rich sequences in the minor groove of DNA (Cattaruzzi *et al*., 2007; Pfannkuche *et al*., 2009; Reeves and Nissen, 1990), and this causes DNA conformational changes to facilitate transcriptional regulation. Under chemotherapeutic stress, these AT‐hooks promote cytoprotective DNA base excision repair (BER) by virtue of their apurinic/apyrimidinic (AP) lyase and 5‐deoxyribose phosphate (dRP) lyase activities (Summer *et al*., 2009). The anti‐apoptotic function of HMGA2 includes alterations in the Ataxia telangiectasia mutated (ATM) and ATM‐Rad3‐related kinase (ATR) DNA damage repair signaling pathways to increase cell survival under genotoxic stress (Natarajan *et al*., 2013; Palmieri *et al*., 2011). HMGA2 also serves as chaperone to protect stalled replication forks from endonucleolytic collapse, thus preventing DNA breaks and promoting replication restart (Yu *et al*., 2014).

In this study, we have identified a novel role of HMGA2 in enhancing DNA damage‐induced PARP1 activity and diminishing the sensitivity of cancer cells to the PARP inhibitor olaparib. Our results suggest that HMGA2 is a predictor for the tumor response to PARP inhibitors and is an attractive therapeutic target for combination therapies using DNA damaging drugs or radiation and PARP inhibitors.

## 2. Materials and methods

### 2.1. Cell lines

The human triple‐negative breast cancer cell lines MDA‐MB‐231, MDA‐MB‐436, the stable MDA‐MB‐231‐HMGA2 transfectant with exogenous expression of full‐size HMGA2 and its vector control MDA‐MB‐231‐mock, the stable UTC8505‐HMGA2 transfectant (Natarajan *et al*., 2016), the HT1080‐C1 fibrosarcoma cell line with doxycycline‐inducible pTRIPz‐shHMGA2 (Origene) (Natarajan *et al*., 2013; Yu *et al*., 2014), and PARP1 wild‐type (MEF^*Parp1*+/+^) and *Parp1* knockout MEF cells (MEF^*Parp1*−/−^) (Wang *et al*., 1997) were employed in the study. MDA‐MB‐231 and MDA‐MB‐436 were purchased from ATCC, and the HT1080‐C1 fibrosarcoma cell line was generously provided by P. Droege (Nanyang University, Singapore) and authenticated by STR profiling (100% identity) at the University of Arizona Genetics Core. Cells were cultured in DMEM/F12 1:1 (Thermo Fisher Scientific, Ottawa, Ontario) plus 5% fetal bovine serum (FBS) under 5% CO_2_ at 37 °C. C1 cells and MEF cells were cultured with 10% FBS. Puromycin dihydrochloride (3 µg·mL^−1^; Sigma‐Aldrich, Oakville, ON, USA) was added for C1 cells.

### 2.2. Treatments

AZD2281 (olaparib, cat# 10621‐10) was purchased from Cayman Chemical Co, USA, and the cells were treated 4 h or 24 h prior to incubation with DNA alkylator methyl methanesulfonate (MMS; Sigma‐Aldrich) at the indicated concentrations. PARG inhibitor [6,9‐diamino‐2‐ethoxyacridine lactate monohydrate (DEA), Sigma‐Aldrich] was used at 1 μm for 24 h prior to IP to prevent PAR degradation during PAR‐IP.

### 2.3. HMGA2 silencing

The pTRIPZ vector construct for doxycycline‐induced HMGA2 shRNA (Dharmacon Oligo‐id V2THS_172476) targets the 3′UTR of *HMGA2* mRNA and contains the shRNA sequence ‘TTGAGGTACAGACTTGGAG’. Induction of sh*HMGA2* in C1 cells was achieved with 4 µg·mL^−1^ doxycycline (Dox) for 96 h with a replenishment cycle every 24 h. Knockdown (KD) of endogenous *HMGA2* in MDA‐MB‐231 and MDA‐MB‐436 cells was achieved by treatment with 40 nm of the open reading frame targeting small interference RNA (siRNA) for *HMGA2* (#SASI_Hs01_00098053, sequence ‘GGAAGAACGCGGUGUGUAA(dT)(dT)’) using SilentFect Bio‐Rad, ON, Canada). Nonsilencing scrambled siRNA (#AS022Y9R, Ambion, CA, USA) was used as control.

### 2.4. Induction of PARP1 activity and PARylation detection

Cells were serum starved for 1 h prior to treatment with MMS for the induction of PARP1 activity, and cells were lysed in 4 °C denaturing protein lysis buffer. For PARP inhibition, cells were incubated with AZD2281 (olaparib) for 24 h prior to MMS treatment. For recovery experiments, cells were washed and recovered in serum‐free medium for the indicated times. PARP1 activity was determined by quantitative assessment of PAR residues using western blot and densitometry with beta‐actin as reference.

### 2.5. Immunoblots

Protein sample preparation and electrophoresis were performed as previously described (Natarajan *et al*., 2013). Primary antibodies employed in the study were rabbit polyclonal antibodies to HMGA2 (1 : 1000), PARP1 (1 : 1000), γH2AX (1 : 1000), α‐tubulin (1 : 1000) (all Cell Signaling Technology, Pickering, ON, USA), histone H3 (1 : 5000), and topoisomerase‐I (1 : 2000) (both Abcam, Toronto, ON, USA), and mouse monoclonal antibodies to PAR (1 : 1000) (Tulip Biolabs, Lansdale, PA, USA), lamin A/C (1 : 500), and NAMPT (1 : 500) (both Santa Cruz Biotechnology, Dallas, TX, USA), beta‐actin (1 : 10 000), PARG (1 : 1000), and Flag (1 : 1000) (all Sigma‐Aldrich). Secondary antibodies used were HRP‐conjugated anti‐rabbit IgG, anti‐mouse IgG (both 1 : 2000, Cell Signaling Technology, NEB, Whitby, ON, USA), and anti‐mouse IgG (1 : 5000, Sigma‐Aldrich).

### 2.6. Immunofluorescence

Experiments were carried out as previously described (Natarajan *et al*., 2016). Primary antibodies were HMGA2 (D1A7) (1 : 4000; rabbit monoclonal, Cell Signaling Technology) and PARP1 (1 : 1500; mouse monoclonal, Santa Cruz Biotechnology); secondary antibodies were Alexa Fluor (AF) 488 conjugated anti‐rabbit IgG and AF594 conjugated anti‐mouse IgG (both Life Technology, Burlington, ON, USA). For analysis, 50 nuclei were chosen for each condition, and colocalizing spots were counted using imagej software (https://imagej.nih.gov/ij/) and graphed using Graph pad prism software.

### 2.7. Proximity ligation assay

Proximity ligation assay (PLA) experiments were done using the Duolink kit (Sigma‐Aldrich) as previously described (Yu *et al*., 2014) according to manufacturer's instructions using the red detection reagents and Mouse Minus and Rabbit Plus reagents. Primary antibodies were HMGA2 (D1A7) (1 : 4000; Cell Signaling Technology) and PARP1 (1 : 1000; Santa Cruz Biotechnology). Slides were imaged on a Zeiss Axio Imager. Images were composed of 50 z‐stacks at 0.2 μm thickness. A minimum of 50 nuclei was quantified per treatment group using the duolink image tool software (MilliporeSigma, St. Louis, MO, USA).

### 2.8. Nuclear fractionation for chromatin‐bound and soluble protein fractions

C1 cells with Dox‐inducible sh*HMGA2* were treated with AZD2281 (olaparib) for 4 h prior to exposure to the alkylating drug MMS for 20 min. Cells were harvested immediately after MMS treatment for protein fractionation into chromatin‐bound and soluble nuclear proteins as described previously (Robu *et al*., 2013). Briefly, cells were resuspended for 5 min in whole‐cell lysis buffer containing 10 mm Hepes (pH 7.8) 0.34 sucrose, 10% glycerol, 10 mm KCl, 1.5 mm MgCl2, 1 mm PMSF, 0.1% Triton X‐100, and protease and phosphatase inhibitors. The cytoplasmic fraction was separated from nuclear pellet by centrifugation for 5 min at 4 °C and 1500 g. The nuclear pellet was lysed for 30 min on ice in nuclear lysis buffer containing 50 mm Tris/HCl (pH 7.8), 420 NaCl, 0.34M sucrose, 0.5% IGEPAL, and protease and phosphatase inhibitors. The chromatin fraction was separated from the nucleoplasm by centrifugation for 30 min at 16 000 ***g***, and the pellet was suspended in chromatin lysis buffer containing 20 mm Tris/HCl (pH 7.5), 100 mm KCl, 2 mm MgCl2, 1 mm CaCl2, 0.3M sucrose, 0.1% Triton X‐100, protease inhibitors, phosphatase inhibitors, and 1 mm PMSF and briefly sonicated on ice‐water. The chromatin‐bound proteins were extracted by incubation with micrococcal nuclease (50 U·mL^−1^) for 40 min at RT.

### 2.9. Co‐immunoprecipitation assays

Following treatments as indicated, cells were washed and collected in ice‐cold PBS and pelleted by centrifugation at 500 g. For the immunoprecipitation (IP) of HMGA2 and PARP1, nuclear protein lysates were prepared. Cells were incubated in whole‐cell lysis buffer containing 10 mm HEPES (pH 7.8), 0.34M sucrose, 10% glycerol, 10 mm KCl, 1.5 mm MgCl2, 1 mm PMSF, 0.1% Triton X‐100, and protease and phosphatase inhibitors for 7 min and centrifuged at 2000 ***g*** for 10 min. The nuclear pellet was resuspended in nuclear lysis buffer containing 50 mm Tris/HCl (pH 7.5), 150 mm sodium chloride, 25 mm sodium fluoride, 0.1 mm sodium ortho‐vanadate, 0.2% Triton X‐100, and 0.3% NP‐40 (Kedar *et al*., 2008) containing protease inhibitors. For IP, 100 μg of protein was incubated with anti‐PARP1 antibody and anti HMGA2 antibody (both rabbit polyclonal, Cell Signaling Technologies), respectively, overnight at 4 °C. Then, A/G magnetic beads were added and incubated for 4 h at 4 °C. Beads were washed 3x with lysis buffer, and proteins were eluted using 3x Laemmli buffer (1M Tris/HCl pH 6.8, 20% SDS, glycerol, bromophenol blue, mercaptoethanol SDS elution buffer by boiling at 65 °C for 15 min and used for western blot. HMGA2 IP for PARP1 detection was also performed in the presence of 200 μg·mL^−1^ ethidium bromide (Ethbr). Ethbr was added for 1 h at 4 °C prior to IP and during all washing steps (Robu *et al*., 2013). Prior to IP of PAR and HMGA2, cells were treated with 1 μm PARG inhibitor [6,9‐diamino‐2‐ethoxyacridine lactate monohydrate (DEA)] for 24 h. For IP, cells were lysed with 1 mL of ice‐cold PAR lysis buffer containing (40 mm HEPES pH 7.5, 120 mm NaCl, 0.3% CHAPS, 1 mm EDTA, and protease, phosphatase and PARG inhibitors (Gagne *et al*., 2012). Protein G magnetic beads were incubated with anti‐pADR clone 10H antibody at a concentration of 1 μg·mL^−1^ (Tulip Biolabs, Lansdale, PA, USA) for 45 min at RT, washed with lysis buffer, and incubated with samples for 2 h at 4 °C. Samples were washed thrice and eluted using SDS elution buffer by boiling the beads at 65 °C for 15 min.

### 2.10. Expression of HMGA2 constructs

C1 cells were treated with doxycycline to downregulate endogenous HMGA2. Flag‐tagged *HMGA2* constructs (AT‐hook 1‐3 mutant and full size) cloned into the eukaryotic expression vector pcDNA3.1(+) were transiently transfected in C1 cells using Effectene reagent (Qiagen, Montreal, QC, Canada) and assayed 48 h after transfection. Alanine mutations rendered nonfunctional AT‐hooks (Cattaruzzi *et al*., 2007), and the N‐terminal Myc NLS (PAAKRVKLD) was introduced for nuclear localization of the mutant protein (Fig. S4).

### 2.11. Cell viability and Caspase 3/7 assays

Cells were seeded in 96‐well plates (3 × 10^4^ cells/well), treated with siRNA (scrambled and siHMGA2), and 72 h later exposed to methyl methanesulfonate (MMS) for 20 min. Cells were recovered in medium with or without olaparib for 24 h, WST reagent or luminescent caspase substrate was added, and the relative luminescence was measured using a multiplate reader (Perkin Elmer, Waltham, MA).

### 2.12. Mitochondrial respiration

Cells were treated with nonsilencing si‐control or with si‐HMGA2. 72 h after si‐treatment, cells (6 × 10^4^ per well) were seeded into XF24 V7 multiwell plates. 24 h after seeding, medium was changed to XF assay medium 1 h prior to the assessment of cellular mitochondrial function using the Seahorse XF24 Extracellular Flux Analyzer (Agilent, Mississauga, ON, Canada) as previously described (Nguyen *et al*., 2016) to assess mitochondrial function.

### 2.13. Statistical analysis

Student's t‐test and one‐way analysis of variance (ANOVA) were used to determine the significance between the groups. Tukey's multiple comparisons test was used between the groups, and a *P*‐value < 0.05 was considered statistically significant.

## 3. Results

### 3.1. HMGA2 increases PARP1 activity upon alkylating DNA damage

**Figure 1:**
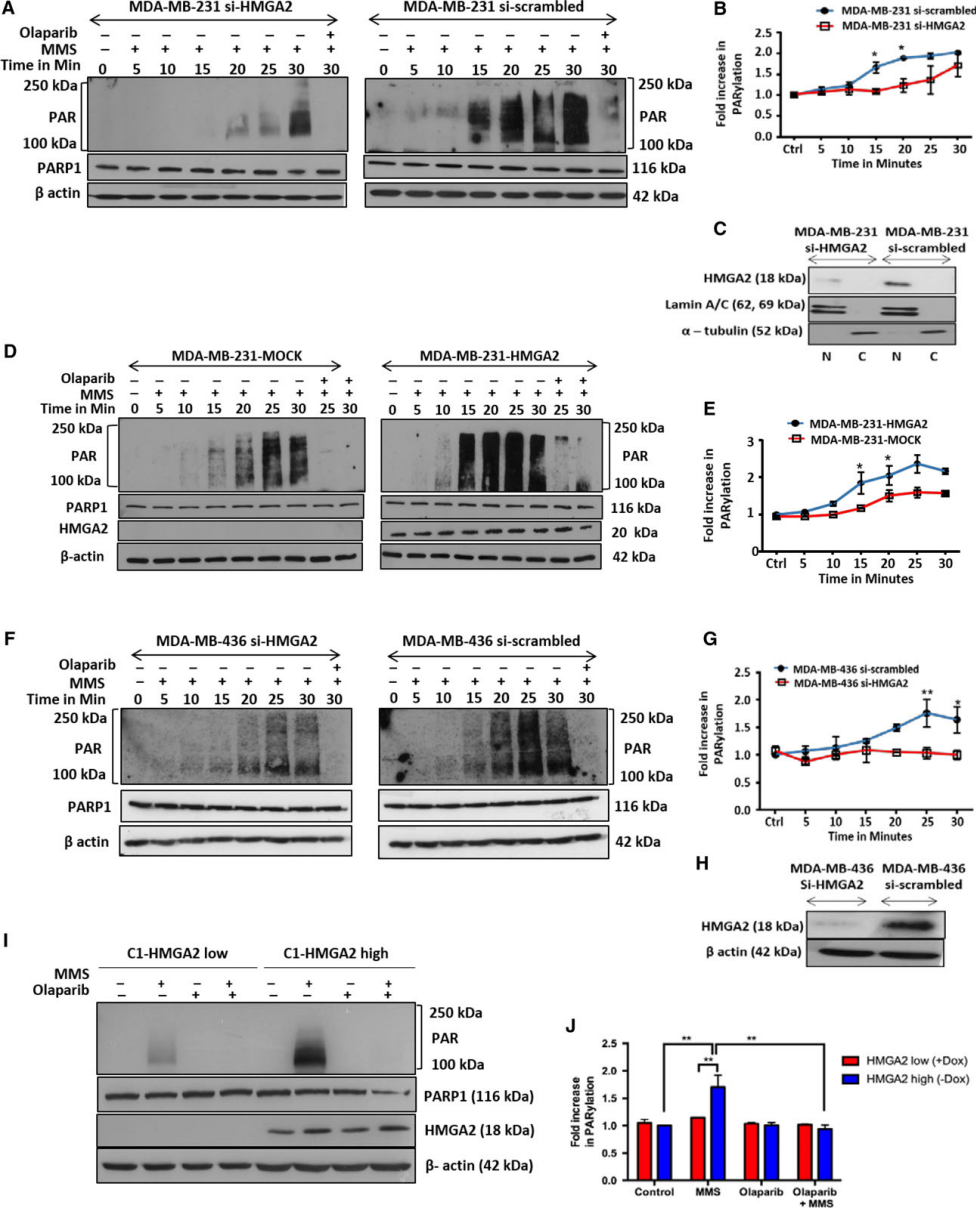
High‐mobility group A2 increases DNA damage‐induced PARP‐1 activity (PARylation) in TNBC and C1 fibrosarcoma cells. Western blot detection of PAR was performed in TNBC cells treated with 4 mm MMS at indicated times. (A) MDA‐MB‐231 cells showed later onset and decreased PARylation following siRNA‐mediated HMGA2 KD compared to cells with endogenous HMGA2. (C) Representative WB of nuclear lysates from MDA‐MB‐231 cells showing downregulation of HMGA2 upon si*HMGA2* treatment compared to si‐scrambled. (D) MDA‐MB‐231 HMGA2 overexpressing cells treated with 4 mm MMS showed increased and early onset of PARylation compared to the mock controls. Note: The low levels of endogenous HMGA2 protein from total cell lysates in MDA‐MB‐231‐Mock cells are not detected in this WB (see Suppl. Fig. 1B for nuclear protein fractions). (F) Similarly, MDA‐MB‐436 cells with endogenous HMGA2 levels showed earlier and increased PARylation upon MMS treatment compared to MDA‐MB‐436 cells upon HMGA2 KD. (H) Representative WB showing HMGA2 KD upon siHMGA2 treatment in MDA‐MB‐436. (I) Representative WB blot for PAR detection in C1 cells upon treatment with olaparib (20 μm), MMS (4 mm), and doxycycline (Dox)‐mediated HMGA2 KD. PARP1 protein levels remained unchanged upon HMGA2 KD. (B, E, G, J) PAR detection was quantified by densitometry, normalized to the respective β‐actin signals, and presented as PARylation from *n* = 3 independent experiments. PAR levels of MMS‐treated C1 cells with low HMGA2 levels were set as 1. One‐way ANOVA and Tukey's multiple comparisons test were performed to determine significance. Data were shown as mean ± SEM; *P <* *0.05; **P <* *0.01.

Methyl methanesulfonate‐induced alkylating DNA damage triggers PARP1 activation which results in an early and transient poly‐ADP ribosylation (PARylation) of proteins. We determined the effect of HMGA2 on the kinetics and intensity of protein PARylation in the HMGA2 expressing cell lines (Fig. S1). Quantitative western blot analysis of cellular lysates collected at 5‐min intervals during short‐term exposure (0‐30 min) of cells with MMS revealed a delayed onset and lower intensity in the level of total PARylated cellular proteins (Fig. 1A,B) upon HMGA2 KD in MDA‐MB‐231 (Fig. 1C). This newly discovered function of HMGA2 in promoting protein PARylation was confirmed in MDA‐MB‐231 overexpressing HMGA2 which showed a significant increase in PARylated proteins when compared to mock controls (Fig. 1D,E) and in MDA‐MB‐436 breast cancer cells following siRNA‐mediated silencing of HMGA2 (Fig. 1F,G).

Similarly, C1 cells with endogenous HMGA2 showed a significant reduction in protein PARylation following Dox‐induced *HMGA2* KD (Fig. 1I,J), suggesting that the PARylation‐promoting function of HMGA2 was not restricted to TNBC but applicable to a broader range of human tumors. Silencing of *HMGA2* by siRNA or induction of shRNA did not affect cellular levels of PARP1 (Fig. 1A,D,F,I) or PARP2 (Fig. S2) and was specific for HMGA2 as the protein levels for the structurally related HMGA1 remained unchanged (Fig. S2). Next, we used MDA‐MB‐231 mock and HMGA2 overexpressing stable transfectants to address whether HMGA2 can alter the kinetics of de‐PARylation. After a 30‐min exposure to MMS, the alkylating agent was removed and cell lysates collected at defined time points during the recovery period demonstrated that although HMGA2 overexpressing MDA‐MB‐231 cells showed stronger protein PARylation, the level of PARylated protein in both, mock and HMGA2 transfectants, became undetectable at approx. 25 min (Fig. S3A,B). We concluded that HMGA2 levels do not alter the kinetics of de‐PARylation.

### 3.2. HMGA2 increases tumor cell resistance to olaparib

**Figure 2:**
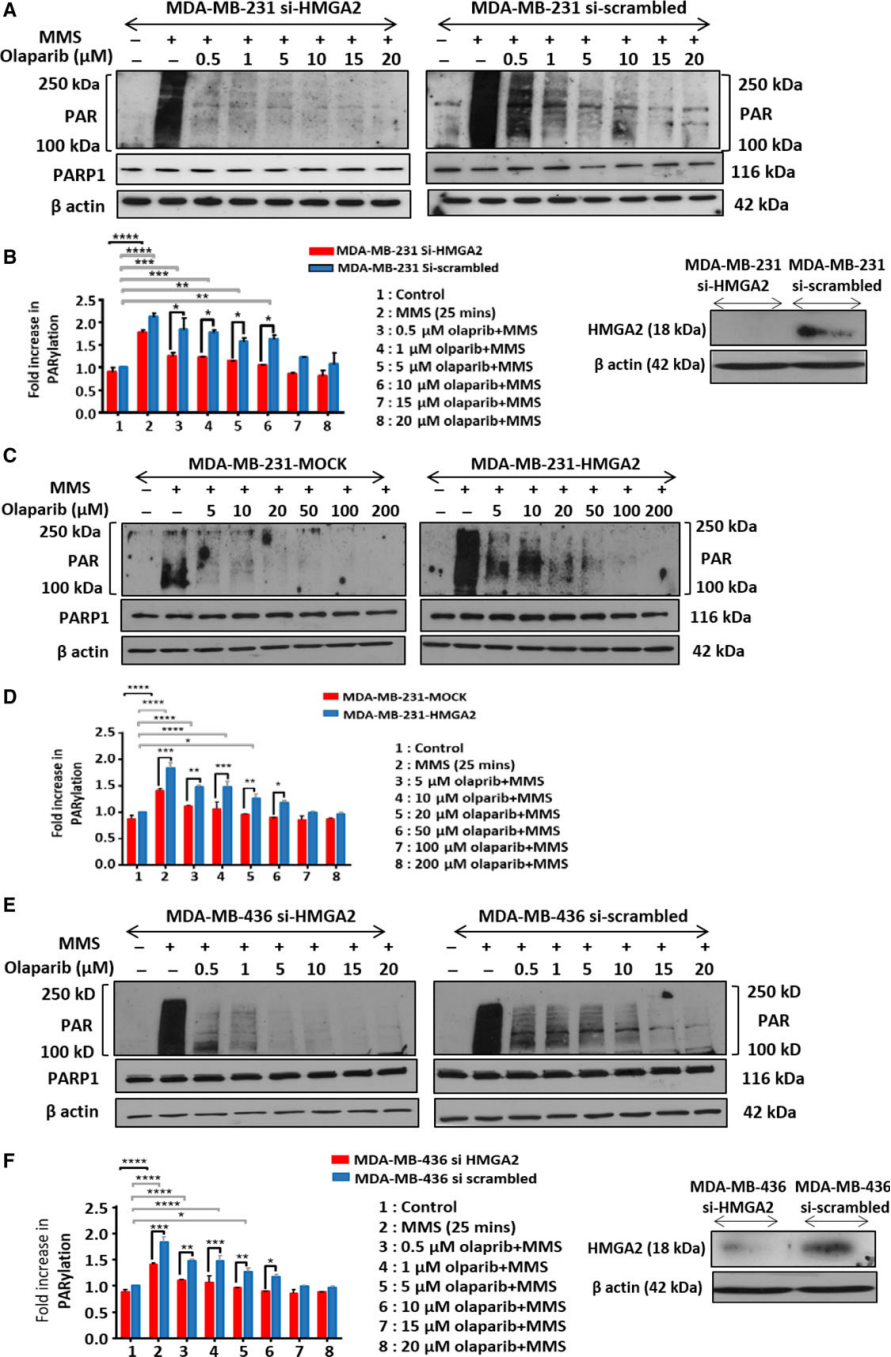
High‐mobility group A2 decreases olaparib sensitivity in TNBC cells. MDA‐MB‐231 and MDA‐MB‐436 cells were treated with olaparib at concentrations ranging from 0.5 to 20 μm or to 200 μm. High cellular HMGA2 levels required increased olaparib concentrations to inhibit DNA damage‐induced PARylation activity. Representative WBs are shown for (A) MDA‐MB‐231 cells with siRNA‐mediated HMGA2 KD, (C). MDA‐MB‐231‐Mock and MDA‐MB‐231‐HMGA2 clones, and (E) MDA‐MB‐436 cells with siRNA‐mediated HMGA2 KD. Insets show downregulation of HMGA2 upon siHMGA2 compared to si‐scrambled by WB of total lysates from MDA‐MB‐231 cells and MDA‐MB‐436. (B, D, F) PAR was quantified by densitometry analysis from three independent experiments, normalized with corresponding actin controls, and presented in the graph as PARylation compared to MMS untreated (control) cells. One‐way ANOVA and Tukey's multiple comparisons test were performed to determine significance from *n* = 3 independent experiments. Data were shown as mean ± SEM. *P <* *0.05; **P <* *0.01; ***P <* *0.001, ****P <* *0.0001.

The presence of HMGA2 enhanced DNA damage‐induced PARP1 activation (Fig. 1), suggesting that higher concentrations of PARP1 inhibitor olaparib may be required to block PARP1 activity in HMGA2 expressing tumor cells. To test this, we quantified PARylation in our tumor cell models with increasing concentrations of olaparib. Upon MMS challenge, five times higher concentrations of olaparib were required to block PARylation in the presence of endogenous HMGA2 compared to HMGA2‐silenced cells in MDA‐MB‐231 (Fig. 2A,B) and MDA‐MB‐436 (Fig. 2E,F). Consistent with this, PARylation in MDA‐MB‐231 mock transfectants was blocked at around 10‐fold lower olaparib concentrations when compared to the corresponding HMGA2 overexpressing transfectants (Fig. 2C,D). Thus, the cellular HMGA2 protein levels determined the efficacy of PARP inhibitor olaparib in blocking PARP activity in human tumor cells.

### 3.3. Functional AT‐hooks of HMGA2 are required to promote PARylation

**Figure 3:**
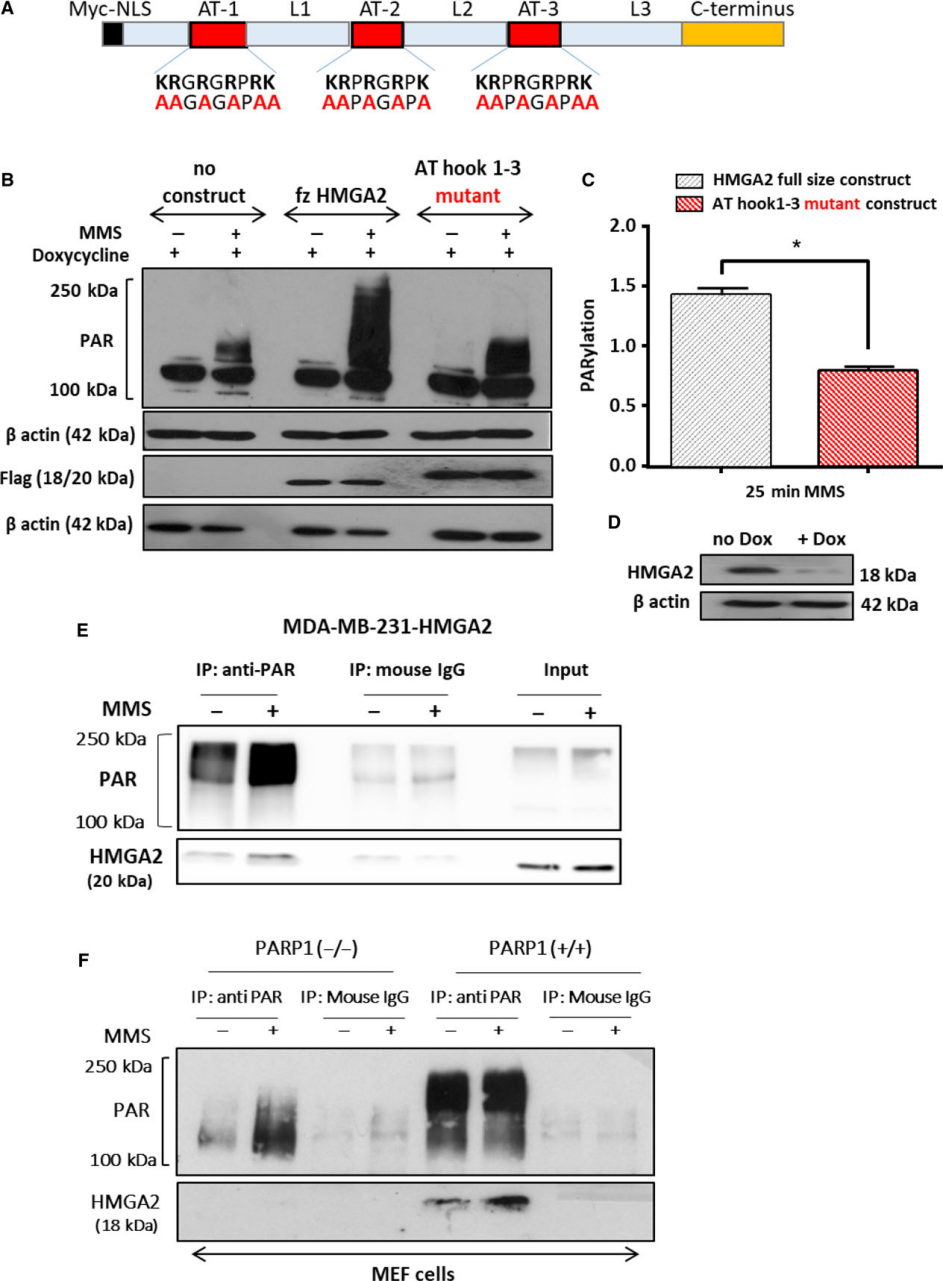
High‐mobility group A2 AT‐hook DNA‐binding domains are essential for HMGA2‐mediated increased PARP1 activity and HMGA2 is PARylated upon DNA damage. (A) Schematic presentation of the N‐terminal Myc nuclear localization domain and the HMGA2 AT‐hook domains with the position of alanine residues in the mutant construct. (B) Representative WB showing MMS induced PARylation in C1 cells after Dox mediated downregulation of endogenous HMGA2 and transient transfection of the wild‐type and AT1‐3 mutant HMGA2 constructs. Exogenous HMGA2 was detected with anti‐Flag antibody, and β‐actin served as protein loading control. (C) PAR was quantified by densitometry, normalized to corresponding actin and flag controls, and presented in the graph as PARylation. Data are shown as mean ± SEM. *P < 0.05 was considered significant by Student's t‐test from *n* = 3 independent experiments. (D) WB showing successful downregulation of endogenous HMGA2 upon Dox treatment. **(**E) MDA‐MB‐231 cells overexpressing HMGA2 were treated with MMS (4 mm) for 30 min, and total protein lysates were immunoprecipitated with an anti‐PAR antibody. PAR was increased following MMS treatment, and HMGA2 was co‐immunoprecipitated with PAR. (F) PARP1 wild‐type MEF (PARP1+/+) cells, but not PARP1‐KO MEFs (PARP1−/−), showed HMGA2 PARylation. Mouse IgG used as a control did not show any pull‐down of PAR or HMGA2.

To determine the structural requirements for the PARylation‐promoting function of HMGA2, we transiently transfected full‐size Flag‐tagged HMGA2 and a Flag‐tagged HMGA2 AT‐hook 1‐3 mutant expression construct in Dox‐treated C1 cells (HMGA2^low^) (Fig. 3A) (Cattaruzzi *et al*., 2007). Basic residues within the second AT‐hook determine the nuclear localization signal (NLS) important for the nuclear compartmentalization of HMGA2 (Cattaruzzi *et al*., 2007). To ensure that HMGA2 mutants with nonfunctional alanine‐mutated AT1‐3 hooks would localize exclusively to the nuclear compartment, we included an N‐terminal c‐Myc NLS sequence. Flag immunofluorescence confirmed the nuclear localization and revealed 16‐30% transient transfection efficiency in Dox‐treated C1 cells (Fig. S4). Residual PARylation detected in untransfected Dox‐treated C1 cells (HMGA2^low^) (Fig. 3B) reflected successful KD of endogenous HMGA2 (Fig. 3D) and marked the baseline levels of PARylation. Strongest PARylation was detected with the full‐size *HMGA2* construct (Fig. 3B,C). Expression of an *HMGA2* construct with the AT1‐3 hooks mutated showed substantially reduced DNA damage‐induced PARylation compared to the full‐size HMGA2 (Fig. 3B,C). In conclusion, the increased protein PARylation required the presence of functional AT1‐3 hooks of HMGA2.

### 3.4. HMGA2 is a target for PARylation by PARP1

To determine whether HMGA2 was a target of PARylation, we treated our tumor cell models with MMS and performed co‐immunoprecipitation studies with an antibody specific for poly‐ADP‐ribosylated sites. We detected PARylated HMGA2 in the PARylated protein fraction of MDA‐MB‐231 transfectants with stable expression of exogenous *HMGA2* (Fig. 3E) and in MDA‐MB‐436 and C1 fibrosarcoma cells (data not shown), both with endogenous HMGA2 expression, indicating that HMGA2 is PARylated upon PARP activation. We used *Parp*(−/−) MEF cells to determine whether the PARylation of HMGA2 was mediated by PARP1 activation. We confirmed the lack of PARP1 protein and the presence of PARP2 and HMGA2 proteins in *Parp*(−/−) MEF cells (Fig. S5). Upon MMS‐induced DNA damage, we were unable to detect PARylated HMGA2 in *Parp1* knockout MEFs but readily showed HMGA2 PARylation in wild‐type MEF cells (Fig. 3F) demonstrating that PARP1 was responsible for DNA damage‐induced PARylation of HMGA2 in our tumor cell models.

### 3.5. HMGA2 is an interaction partner of PARP1

**Figure 4:**
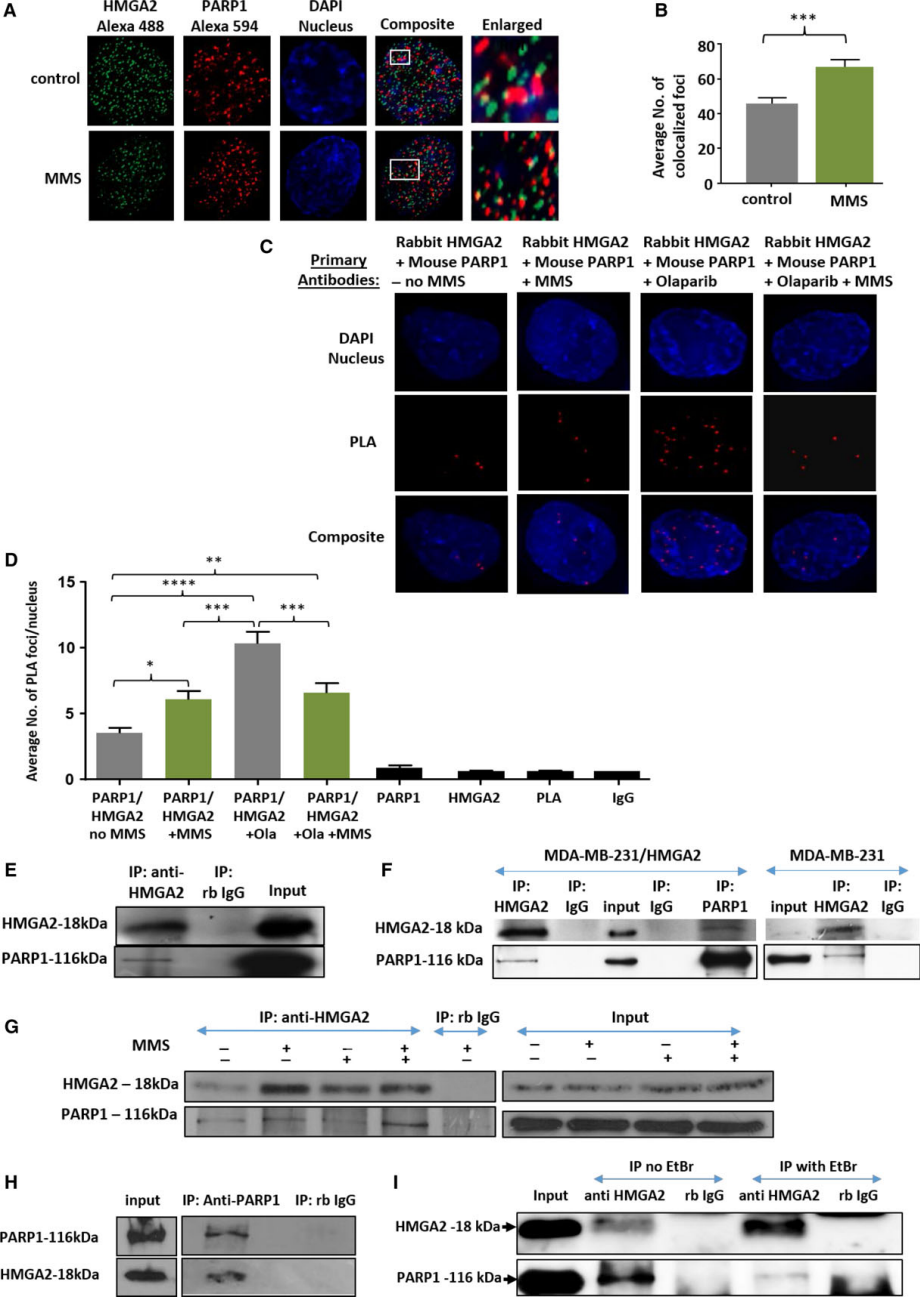
High‐mobility group A2 colocalizes and interacts with PARP1. (A) Representative fluorescence images of single nuclei (C1 cells) are shown (DAPI‐nucleus; red‐PARP; green‐HMGA2). Cells were treated with 5 mm MMS for 15 min. (B) The average number of colocalized foci from a total of 30 nuclei per treatment group was quantified per experiment and graphed from three independent experiments (90 nuclei in total) with error bars representing the mean ± SEM; one‐way ANOVA and Tukey's multiple comparisons test were performed to determine significance; ***P < 0.001. (C) Representative fluorescence images of single nuclei (C1 cells) are shown (DAPI‐nucleus; red‐PLA foci). Cells were treated with 20 μm olaparib overnight and 5 mm MMS for 15 min. (D) The average number of PLA foci per nucleus from a total of 50 nuclei per treatment group was quantified per experiment and graphed from three independent experiments with error bars representing SEM. Primary antibody alone and PLA probe alone served as negative control for the assay (shown in Fig. S6). Data are shown as mean ± SEM. One‐way ANOVA and Tukey's multiple comparisons test were performed to determine significance; ****P < 0.0001, ***P < 0.001, **P < 0.01, *P < 0.05. Co‐IP was performed from nuclear protein extracts using rabbit anti‐HMGA2 and anti‐PARP1 antibodies or a rabbit IgG isotype control. Representative WBs from HMGA2 IPs are shown for the detection of HMGA2 and PARP1 from the input and the precipitated proteins (E) in fibrosarcoma cells (C1) with endogenous HMGA2 expression and (F) in MDA‐MB‐231 with endogenous and overexpressing levels of HMGA2. (G) C1 cells treated with olaparib (20 μm) for 24 h also showed pull‐down of PARP‐1 with HMGA2. Rabbit IgG controls did not show pull‐down of either protein. 20 μg of nuclear proteins was used as loading controls (input) in immunoblots. (H) Nuclear protein extracts from C1 cells were used for the reversed IP using PARP1 antibody and detection for HMGA2. (I) HMGA2 IP performed in the presence of 200 μg/mL ethidium bromide caused an attenuated PARP1 protein pull‐down.

The PARylation of HMGA2 suggested a physical interaction with PARP1, and this was confirmed in fluorescence co‐immunodetection of HMGA2 and PARP1 in C1 cells (Fig. 4A,B). The number of colocalizing HMGA2‐PARP1 foci increased further by up to 35% in the presence of MMS (Fig. 4B). We performed proximity ligation assays (PLA) to determine HMGA2 and PARP1 colocalization within a 40 nm radius from each other (Soderberg *et al*., 2006). Similar to the co‐immunodetection results, PLA detected a preexisting HMGA2‐PARP1 complex in untreated C1 cells and showed an increase in HMGA2‐PARP1 interaction in C1 cells upon DNA alkylation. PARP1 inhibitor olaparib did not affect PLA foci formation under MMS (Fig. 4C,D). By contrast, olaparib alone consistently caused a more than 45% increase in PLA foci when compared to untreated controls or cells exposed to MMS and dual MMS/olaparib treatment (Fig. 4C,D). PLA control experiments with single antibodies gave negligible number of foci (Fig. S6). HMGA2‐PARP1 PLA foci were also detected in MDA‐MB‐231 (Fig. S6B) and MDA‐MB‐436 cells (data not shown).

High‐mobility group A2 co‐immunoprecipitated PARP1 in endogenous HMGA2 producing C1 cells (Fig. 4E) and in MDA‐MB‐231 cells with endogenous HMGA2 levels and in HMGA2 expressing stable transfectants (Fig. 4F). Immunoprecipitation (IP) of HMGA2 in C1 cells treated with MMS, olaparib, and the combination of MMS/olaparib resulted in the successful co‐IP of PARP1 in all treatment groups, with MMS/olaparib cotreatment consistently showing stronger PARP1 co‐IP (Fig. 4G). Reverse IP of PARP1 in MMS/olaparib treated C1 successfully co‐immunoprecipitated HMGA2 (Fig. 4H). Thus, HMGA2 associates with and was a target of PARP1 in the nucleus. HMGA2 IP performed in the presence of ethidium bromide yielded a weaker PARP1 pull‐down (Fig. 4I), indicating that the association of PARP1 with DNA promotes the HMGA2‐PARP1 interaction.

### 3.6. HMGA2 reduces olaparib‐induced PARP1 trapping to MMS‐damaged DNA

**Figure 5:**
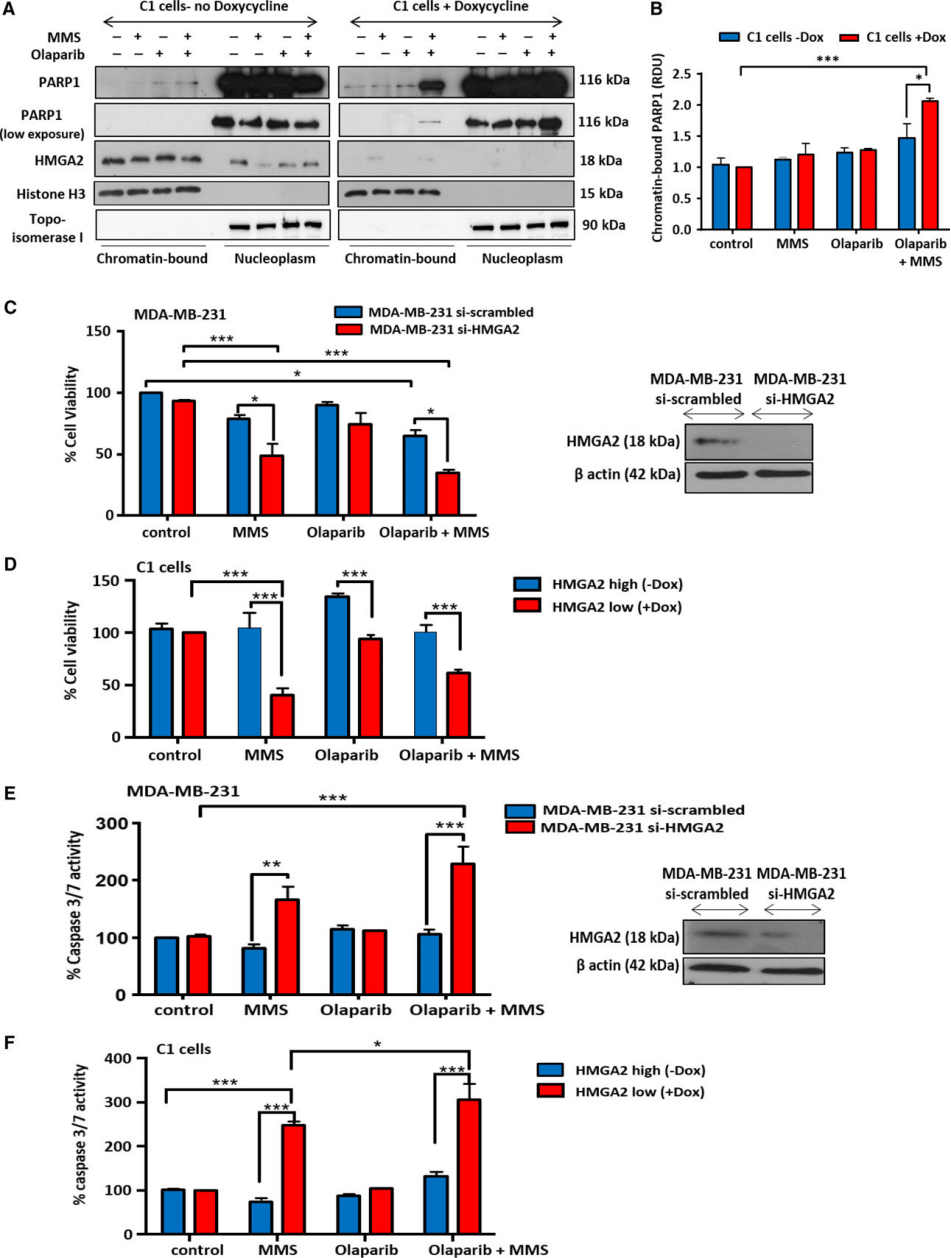
High‐mobility group A2 prevents PARP1 trapping, increases cell viability, and reduces apoptosis following DNA damage and olaparib treatment. (A) Representative WB using chromatin‐bound and soluble (nucleoplasm) nuclear protein fractions of C1 cells to localize PARP1 protein after treatment with olaparib and MMS. C1 cells were cultured without (HMGA2^high^) or with Dox (HMGA2^low^) and exposed to olaparib and MMS as indicated. Histone H3 and topoisomerase‐I were detected to confirm equal protein loading for chromatin‐bound and soluble nuclear fractions, respectively. (B) Chromatin‐bound PARP1 was quantified by densitometry, normalized to the respective histone H3 signals, and presented relative to the values of chromatin‐bound PARP1 in untreated controls which were set as 1. Data were shown as mean ± SEM; one‐way ANOVA and Tukey's multiple comparisons test were performed to determine significance; *P < 0.05; **P < 0.01, ***P < 0.001. (C, E) MDA‐MB‐231 and (D, F) C1 cells were treated with olaparib (20 μm) for 24 h prior to MMS treatment (4 mm, 30 min), and cell viability was determined after 24 h by WST assay. (C) In MDA‐MB‐231 cells, MMS‐induced DNA damage reduced cell viability only with HMGA2 silencing and PARP inhibition aggravated this effect. (D) Similarly, in C1 cells HMGA2 silencing reduced cell viability following 4 mm MMS alone or combined MMS and olaparib (20 μm) treatment. (E) In MDA‐MB‐231 cells, MMS‐induced DNA damage only activated apoptosis when HMGA2 was silenced. PARP inhibition did not further increase caspase 3/7 activity. (F) Similarly, in C1 cells HMGA2 silencing was required to induce caspase 3/7 activity with 4 mm MMS alone or after combined MMS and olaparib (20 μm) treatment. Framed insets show representative WBs for siRNA‐mediated HMGA2 KD in MDA‐MB‐231 cells. The values obtained by colorimetric quantification in the untreated controls were set to 100%. Data are presented as % cell viability or % caspase 3/7 activity compared to MMS untreated cells. Data were shown as mean SEM ± from three independent experiments. One‐way ANOVA and Tukey's multiple comparisons test were performed *P < 0.05; **P < 0.01; ***P < 0.001.

We also tested the ability of HMGA2 to affect the recently described cytotoxic function of olaparib in trapping PARP1 to DNA damage sites (Kedar *et al*., 2012; Murai *et al*., 2014; Shen *et al*., 2015). Following Dox‐induced knockdown of HMGA2 in C1 cells, we performed treatments with MMS or olaparib alone and in combination prior to extraction of chromatin‐bound and soluble nuclear proteins. Silencing of HMGA2 markedly increased chromatin‐bound PARP1 following combined MMS treatment and PARP inhibition (Fig. 5A,B). We conclude that HMGA2 prevents olaparib‐induced trapping of PARP1 at DNA damaged sites. Indeed, combined HMGA2 silencing with PARP1 inhibition under MMS was accompanied by increased double‐strand breaks (DSB) as determined by the detection of DSB marker γH2AX after 24 h (Fig. S7). Nuclear co‐immunofluorescence for PARP1 and γH2AX revealed more colocalized foci exclusively under HMGA2 KD (Fig. S8A). The increased size of γH2AX foci (Fig. S8B*c*) and the increased average integrated fluorescence intensity of γH2AX signals (Fig. S8C) upon olaparib and MMS cotreatment in HMGA2 KD cells confirmed enhanced PARP1 DNA trapping and DNA damage.

### 3.7. Cytoprotective role of HMGA2 under PARP inhibition and alkylating DNA damage

The novel role of HMGA2 in enhancing PARP1 PARylation activity and reducing efficacy of olaparib in blocking PARP1 function and PARP1 DNA trapping is expected to impact on tumor cell viability. To test this, we assessed the response of TNBC and fibrosarcoma cells to *HMGA2* KD and treatment with MMS, olaparib, and combined MMS/olaparib. While single treatments with MMS or olaparib failed to reduce cell viability over 24 h in MDA‐MB‐231 and C1 cells, the silencing of endogenous *HMGA2* significantly reduced cell viability with MMS treatment (Fig. 5C,D). Combined MMS/olaparib treatment was significantly more toxic after HMGA2 silencing, resulting in a less than 40% cell viability compared to more than 60% cell viability in the presence of HMGA2 (Fig. 5C,D). In agreement with the cytotoxicity data, *HMGA2* silencing was essential to induce caspase 3/7 activation in MDA‐MB‐231 cells (Fig. 5E), C1 cells (Fig. 5F) and MDA‐MB‐436 cells (Fig. S9) upon MMS or combined MMS/olaparib treatment. As a potent antagonist of PARP inhibitor function, the cytoprotective role of HMGA2 may contribute to reduced efficacy of PARP inhibitors in HMGA2‐positive tumors.

### 3.8. HMGA2 expressing TNBC cells have higher protein levels of nicotinamide phosphoribosyltransferase and higher mitochondrial respiratory capacity

**Figure 6:**
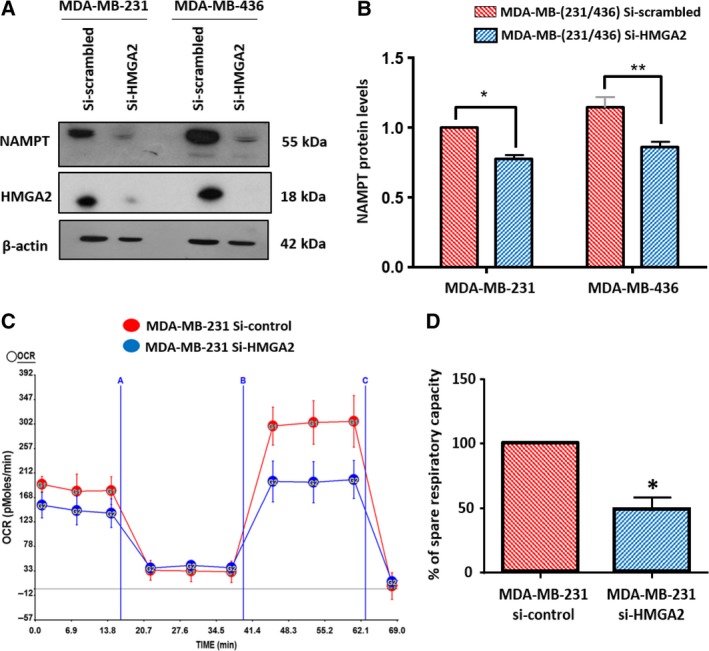
High‐mobility group A2 KD decreases NAMPT protein levels and reduces the mitochondrial respiratory capacity. MDA‐MB‐231 and MDA‐MB‐436 cells were treated with scrambled siRNA or with siHMGA2. **(**A) Representative WB showing reduced NAMPT protein levels following siRNA‐mediated HMGA2 KD in both cell lines. (B) NAMPT protein levels were quantified by densitometry in relation to β‐actin, and the endogenous protein expression of NAMPT in MDA‐MB‐231 cells was set to 1 in the graph. C**.** Oxygen consumption rates (OCR) in MDA‐MB‐231 cells were measured in real time under baseline conditions and after injection of 1 μm oligomycin (A), 0.75 μm FCCP (B), and 1 μm rotenone plus antimycin (C) as indicated, using the Seahorse XF24 Extracellular Flux Analyzer. (D) Quantitative analysis revealed a reduced spare respiratory capacity under HMGA2 silencing. Data were shown as mean SEM ± from three independent experiments. One‐way ANOVA and Tukey's multiple comparisons test were performed; *P < 0.05; **P < 0.01.

Despite increased PAR consuming PARP1 activity upon DNA damage induction, we observed increased cell viability and reduced apoptosis in HMGA2 expressing cells. We questioned whether increased ADP‐ribose consumption was compensated for by increased replenishing of ADP‐ribose through the salvage pathway (Garten *et al*., 2015). In the presence of HMGA2, we consistently detected higher protein levels of nicotinamide phosphoribosyltransferase (NAMPT) which is the rate‐limiting enzyme of the NAD salvage pathway (Fig. 6A,B). Furthermore, we measured a higher mitochondrial oxygen consumption rate and a higher mitochondrial spare respiratory capacity in the presence of endogenous HMGA2 levels as compared to cells with siRNA‐mediated *HMGA2* KD (Fig. 6C,D). These findings suggest novel metabolic functions of HMGA2 directed at replenishing cellular NAD+ through the salvage pathway and enhancing mitochondrial respiratory reserve, bestowing HMGA2 expressing cancer cells with enhanced mitochondrial and redox capacity.

## 4. Discussion

In the present study, we have identified the chromatin‐binding protein HMGA2 as a new endogenous modulator of PARP1 activity causing accelerated and enhanced PARylation upon DNA damage in human tumor cells. Of note, the presence of HMGA2 itself failed to trigger PARylation without DNA stress. PARylation is a spatially and temporally regulated post‐translational modification involved in the signaling of DNA damage detection and repair, cell death pathways, and transcriptional activity (Kim *et al*., 2005; Li and Chen, 2014). PAR automodifications of PARP1 serve as scaffold for the recruitment of DNA repair factors to the site of damage. The increasing negative charges of PARylated PARP1 result in the removal of PARP1 from the DNA to facilitate DNA damage repair (Luo and Kraus, 2012). The HMGA2‐mediated increase of PAR was undetectable 30 min after MMS treatment. This suggests that PARG and/or other de‐PARylating enzymes have sufficient capacity in cancer cells to warrant the timely degradation of PAR (Alvarez‐Gonzalez and Althaus, 1989). Since 90% of PAR chains are linked to PARP1 itself, the timely de‐PARylation kinetics of automodified PARP1 suggest that its functions in DNA repair are not compromised in the presence of HMGA2. Malignant tumors, including triple‐negative breast cancer cells, re‐expressing oncofetal HMGA2 frequently develop therapeutic resistance to antitumor treatments and have a poor prognosis (Sun *et al*., 2014; Wend *et al*., 2013). In our cancer cell models, we observed that high cellular HMGA2 protein levels required higher concentrations of the PARP inhibitor olaparib to block DNA damage‐induced PARP1 activity. Thus, HMGA2 reduces the sensitivity of tumor cells toward PARP inhibitors.

We identified HMGA2 as a new interaction partner of PARP1, as a modulator of DNA damage‐induced PARP1 activity, and as an ADP‐ribose acceptor in human tumor cells. Previously, HMGN1 (HMG14) was shown to interact with PARP1 and to promote PARylation activity *in vitro* and *in vivo* (Masaoka *et al*., 2012). We were unable to identify a typical PAR‐binding motif in HMGA2 as demonstrated for the PARP1 interaction partner and ADP‐ribose acceptor MRE11 (Haince *et al*., 2008). In situ proximity ligation assay (PLA) assays detect proteins separated by less than 40 nm and showed nuclear HMGA2‐PARP1 colocalization, likely indicating functional interactions. Co‐IP from nuclear protein lysates confirmed this interaction without exogenous DNA damage. This suggested preexisting HMGA2‐PARP1 protein complexes which were further increased following MMS treatment. PARylation of HMGA2 following alkylating DNA damage provided further evidence for a functional interaction between PARP1 and HMGA2 in fibrosarcoma and breast cancer cells. Interestingly, anti‐PAR IP failed to co‐IP HMGA2 in PARP1‐deficient (*Parp1* ^−/−^) MEF cells compared to wild‐type MEFs (*Parp1* ^+/+^) despite readily detectable protein levels of PARP2 in these MEF cells, indicating that PARP1, not PAR moieties, is the predominant interaction partner for HMGA2.

Poly(ADP‐ribose) polymerase 1 and HMGA2 both participate in base excision repair (BER) of alkylating DNA base lesions. PARP1 serves as immediate sensor of single‐strand breaks (SSB) and binds to SSB intermediates (De Vos *et al*., 2012). This PARP1‐DNA binding leads to conformational changes in the helical subdomain (HD) of the catalytic domain (CAT) resulting in catalytic activity (Langelier and Pascal, 2013; Langelier *et al*., 2011). PARP1 functionally or physically interacts with and PARylates several key factors of the BER pathway (De Vos *et al*., 2012). HMGA2 enhances BER by virtue of the AP‐lyase and dRP‐lyase activities of its AT‐hook domains (Summer *et al*., 2009). HMGA2 AP‐lyase function creates the SSB intermediates which activate PARP1. It is conceivable that preexisting HMGA2‐PARP1 complexes may facilitate the early and strong PARP1 activation by means of AP‐lyase activity of HMGA2 and PARP1 (Khodyreva *et al*., 2010). Functional HMGA2 AT‐hooks are essential for the PARP1 activity enhancing role of HMGA2, indicating an important function for the arginines in the AT‐hooks which convey both, DNA‐binding and AP‐site cleavage (Reeves and Nissen, 1990; Summer *et al*., 2009). Our observation that HMGA2 is PARylated upon MMS treatment warrants further studies to investigate whether this PAR modification influences HMGA2 enzymatic activity. A preexisting nuclear HMGA2‐PARP1 complex in cancer cells may facilitate a state of ‘readiness’ to respond to DNA lesions with early‐onset and efficient single‐strand DNA break repair. In support of this notion, we observed a significant reduction in double‐strand DNA breaks, as determined by the reduced γH2AX levels, in the presence of HMGA2. The HMGA2 chromatin remodeling function may also enhance access of PARP1 to sites of DNA damage. In turn, PARP1‐mediated PARylation leads to a rapid and temporary decondensation of chromatin at the site of damage (Strickfaden *et al*., 2016) which could promote increased DNA accessibility of repair factors, including HMGA2.

Importantly, we found that HMGA2 prevented PARP1 trapping at the chromatin following alkylating damage in the presence of olaparib. PARP trapping to DNA by PARP inhibitors was recently shown to promote their cytotoxic effect (Murai *et al*., 2014; Shen *et al*., 2015). Some of the bulkier PARP inhibitors, namely olaparib, rucaparib, niraparib, and talazoparib, cause conformational changes in PARP1 as they bind to the NAD+ binding pocket of the CAT domain and interact with residues in the adenine–ribose binding pocket, the helical domain (HD), and the D‐loop of PARP1. This reverse‐allosteric interdomain signaling (Shen *et al*., 2015) results in stabilization of PARP1‐DNA complexes (Shen *et al*., 2015) and PARP1 trapping at the site of DNA lesions. This leads to replication fork arrest, double‐strand DNA breaks, and ultimately cell death (Murai *et al*., 2014). It is currently uncertain whether PARP1 trapping at the DNA is exclusively caused by inhibitor‐induced allosteric changes in PARP1 or a consequence of inhibition of PARP1 automodification (Hopkins *et al*., 2015). The ability of HMGA2 to prevent PARP1 trapping to genomic DNA was observed at catalytically inhibitory concentrations of olaparib and, thus, was independent of PARylation activity and PAR‐mediated repulsion of PARP1 from the DNA. One possible mechanism by which HMGA2 prevents PARP1 trapping at the site of DNA damage is its DNA‐binding function and AP‐lyase activity (Summer *et al*., 2009). Because the AT‐hook domains of HMGA2 convey AP‐lyase enzymatic activity (Summer *et al*., 2009) and DNA‐binding (Cattaruzzi *et al*., 2007; Pfannkuche *et al*., 2009), it may be the physical binding of HMGA2 to damaged DNA sites that prevent PARP1 from getting trapped at these sites. The results from our experiments with base‐intercalating ethidium bromide suggest that proper association of PARP1 with DNA promotes the HMGA2‐PARP1 interaction. The chromatin relaxation function of HMGA2 potentially contributes to a reduced trapping through altered PARP1 DNA‐binding kinetics (Catez *et al*., 2004).

Consistent with the reduced olaparib‐induced PARP1 trapping in the presence of HMGA2, silencing *HMGA2* was required to significantly reduce cell viability in our tumor cell models upon olaparib treatment in MMS exposed cells. Strikingly, *HMGA2* silencing was essential to induce caspase 3/7‐activation with combined MMS/olaparib treatment, indicating that *HMGA2* silencing facilitated olaparib‐induced PARP trapping and induced apoptosis. In addition, caspase 3/7‐dependent apoptosis following alkylating DNA damage alone was only induced under *HMGA2* silencing. We have previously associated this anti‐apoptotic role of HMGA2 upon alkylating DNA damage with modulation of the ATR‐Chk1 signaling pathway (Natarajan *et al*., 2013). Here, we show that despite MMS/olaparib cotreatment, *HMGA2* knockdown was required to induce caspase 3/7 activation. Thus, PARP1 trapping‐induced apoptosis is prevented by HMGA2.

We demonstrated increased survival of HMGA2 expressing cancer cells under DNA stress, despite the fact that increased PARP1 activity can potentially cause cell death through NAD+ depletion and energy failure (Bouchard *et al*., 2003). Upon *HMGA2* silencing, we observed reduced protein levels of nicotinamide phosphoribosyltransferase (NAMPT), the rate‐limiting enzyme of the salvage pathway for NAD+ synthesis. Although HMGA2 silencing did not change the steady‐state cellular NAD+ levels in our cell models (data not shown), higher NAMPT protein levels in HMGA2+ cancer cells allow for expedient replenishing of NAD+ substrate to facilitate a strong DNA stress‐induced PARylation response. Although our data do not strongly support a causative link between HMGA2 and NAD+ metabolism, an important role for NAMPT in providing NAD+ substrate for PARP catalytic activity was recently shown as NAMPT inhibition by FK866 enhanced the effectiveness of olaparib treatment in triple‐negative breast cancer xenografts (Bajrami *et al*., 2012) and increased cytotoxicity of temozolomide in combination with BER inhibition in glioblastoma (Goellner *et al*., 2011). Importantly, this novel metabolic effect of HMGA2 also involved an increase in baseline mitochondrial oxygen consumption rate and mitochondrial spare respiratory capacity. This provides cancer cells with the mitochondrial ATP resource essential for efficient and timely repair of DNA damage. This novel finding has important implications in light of the recently described PAR signaling from the nucleus to the cytosol, resulting in the inhibition of glycolysis and loss of ATP (Fouquerel *et al*., 2014). Further studies are currently ongoing to better understand the mechanisms underlying these HMGA2 metabolic effects.

## 5. Conclusion

In conclusion, we have identified HMGA2 as a novel PARP1 interaction partner which promotes enhanced PARylation activity of PARP1 upon DNA damage and increases cell survival. Clinically, this role of HMGA2 translated into enhanced olaparib resistance in tumor cells and highlights the importance of HMGA2 as a novel predictive biomarker for the response to PARP inhibitors in tumor cells.

## Conflict of interest

The authors declare no conflict of interest.

## Author contributions

SHK and TK designed the study and provided conceptual guidance, and SHK coordinated with all coauthors. FK and AKK performed PARylation studies and functional cell assays. MM, SN, and TT performed immunoprecipitations and MEF study experiments. SK and FB did the co‐immunofluorescence and PLA experiments. ML helped with PARP1 knockout MEF cells. FX and GH performed the mitochondrial respiration studies; and ML and GH assisted in manuscript presentation.

## Supporting information


**Fig. S1.** HMGA2 and PARP‐1 are expressed in human breast cancer and fibrosarcoma cell lines.
**Fig. S2.** HMGA1 and PARP2 are not affected under SiHMGA2 treatment.
**Fig. S3.** MMS‐induced cellular PAR levels normalize after 30 min recovery time.
**Fig. S4.** Flag‐immunofluorescence on C1 cells transiently transfected with the AT1‐3 mutant (A, B) or full‐size (C, D) HMGA2 constructs.
**Fig. S5.** PARP1 knock‐out MEF cells (PARP1 −/−) lack PARP1 protein but express PARP2 and HMGA2 proteins.
**Fig. S6.** Proximity ligation assays (PLA) demonstrate HMGA2/PARP1 colocalization in MDA‐MB‐231 cells.
**Fig. S7** HMGA2 knockdown along with inhibition of PARP‐1 activity increases DNA double strand breaks (γH2AX).
**Fig. S8** HMGA2 knockdown increases PARP1 co‐localization with the DNA damage marker γH2AX.
**Fig. S9.** HMGA2 silencing increases apoptosis in MDA‐MB‐436 cells.
