# Supplementary material for: HMGA2 as a functional antagonist of PARP1 inhibitors in tumor cells
Source: Mol Oncol. 2018 Nov 28;13(2):153–70. doi: 10.1002/1878-0261.12390 (PMC6360374; doi:10.1002/1878-0261.12390)
Supplement: Supplementary file 1 — Fig. S1. HMGA2 and PARP‐1 are expressed in human breast cancer and fibrosarcoma cell lines. Fig. S2. HMGA1 and PARP2 are not affected under SiHMGA2 treatment. Fig. S3. MMS‐induced cellular PAR levels normalize after 30 min recovery time. Fig. S4. Flag‐immunofluorescence on C1 cells transiently transfected with the AT1‐3 mutant (A, B) or full‐size (C, D) HMGA2 constructs. Fig. S5. PARP1 knock‐out MEF cells (PARP1 −/−) lack PARP1 protein but express PARP2 and HMGA2 proteins. Fig. S6. Proximity ligation assays (PLA) demonstrate HMGA2/PARP1 colocalization in MDA‐MB‐231 cells. Fig. S7 HMGA2 knockdown along with inhibition of PARP‐1 activity increases DNA double strand breaks (γH2AX). Fig. S8 HMGA2 knockdown increases PARP1 co‐localization with the DNA damage marker γH2AX. Fig. S9. HMGA2 silencing increases apoptosis in MDA‐MB‐436 cells. [file MOL2-13-153-s001.docx]

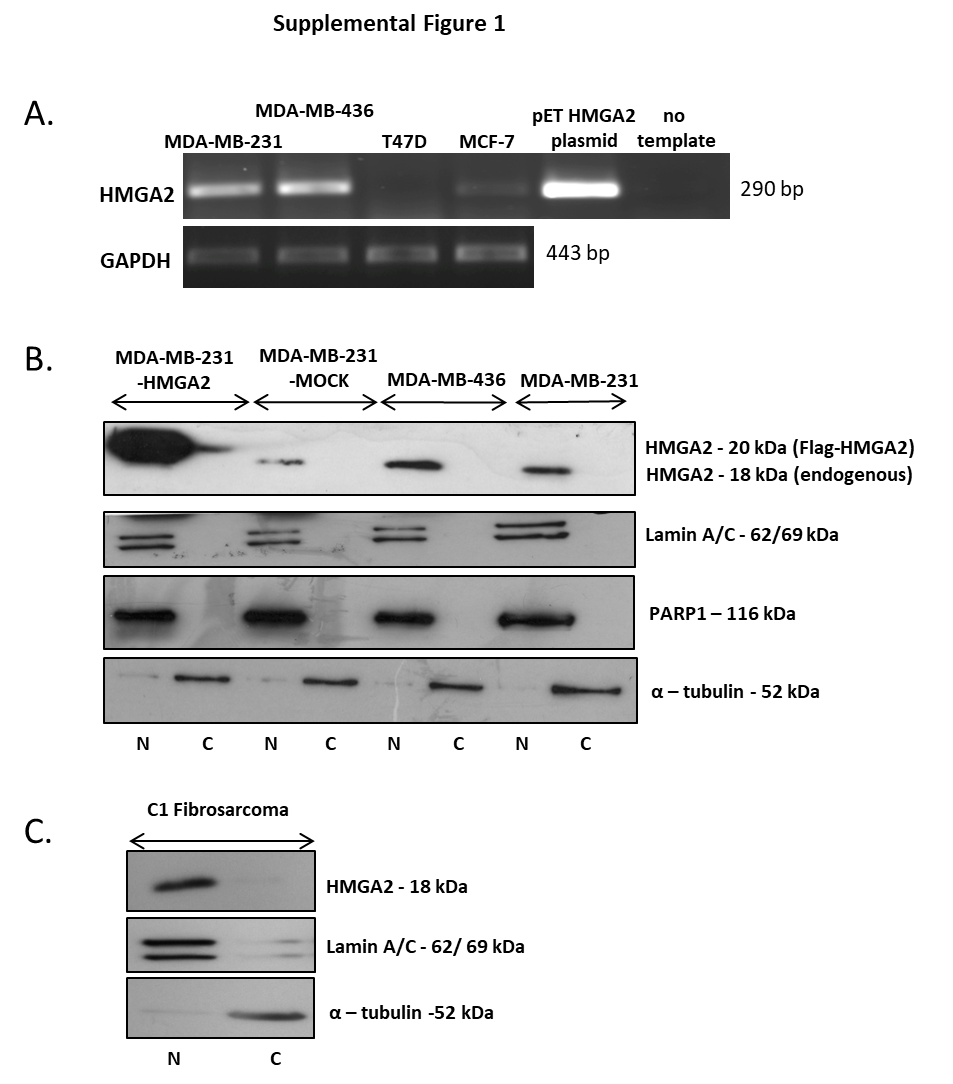


**Suppl. Fig. 1: HMGA2 and PARP-1 are expressed in human breast cancer and fibrosarcoma cell lines. A.** RT-PCR analysis revealed mRNA expression in MDA-MB-231, MDA-MB-436, T47D and MCF-7 cell lines. The pET plasmid encoding for HMGA2 served as positive control. HMGA2 primer sequences: forward 5’-CACTTCAGCCCAGGGACAACC-3’; reverse 5’-CCTCTTCGGCAGACTCTTGTGA-3’. **B., C.** Western blots showing the expression of HMGA2 and PARP-1 protein in the nuclear lysates of MDA-MB-231, MDA-MB-436, HT1080(C1) fibrosarcoma cells. Lamin A/C and α**-**tubulin were used as loading controls for nuclear (N) and cytoplasmic (C) protein fractions, respectively.


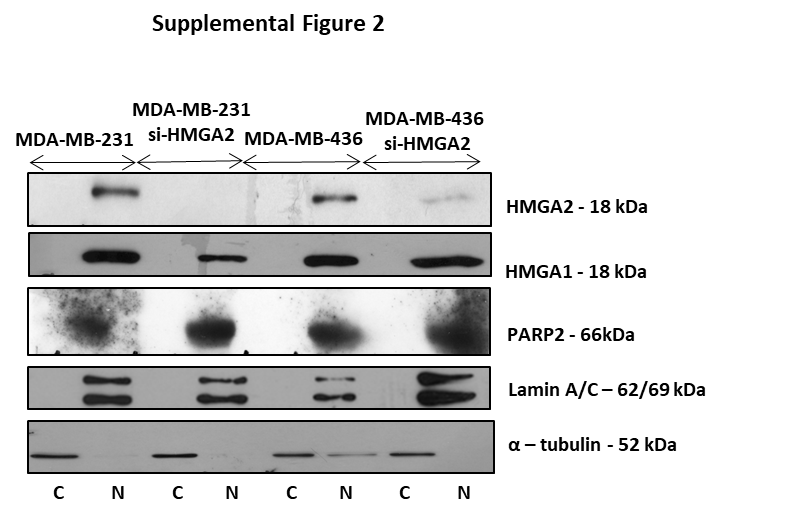


**Suppl. Fig. 2: HMGA1 and PARP2 are not affected under SiHMGA2 treatment.** Representative Western blot showing the expression of HMGA2, HMGA1 and PARP2 protein in nuclear lysates of MDA-MB-231 and MDA-MB-436 cells +/- si-HMGA2 knockdown. Lamin A/C and α**-**tubulin were used as loading controls for nuclear (N) and cytoplasmic (C) protein fractions, respectively.


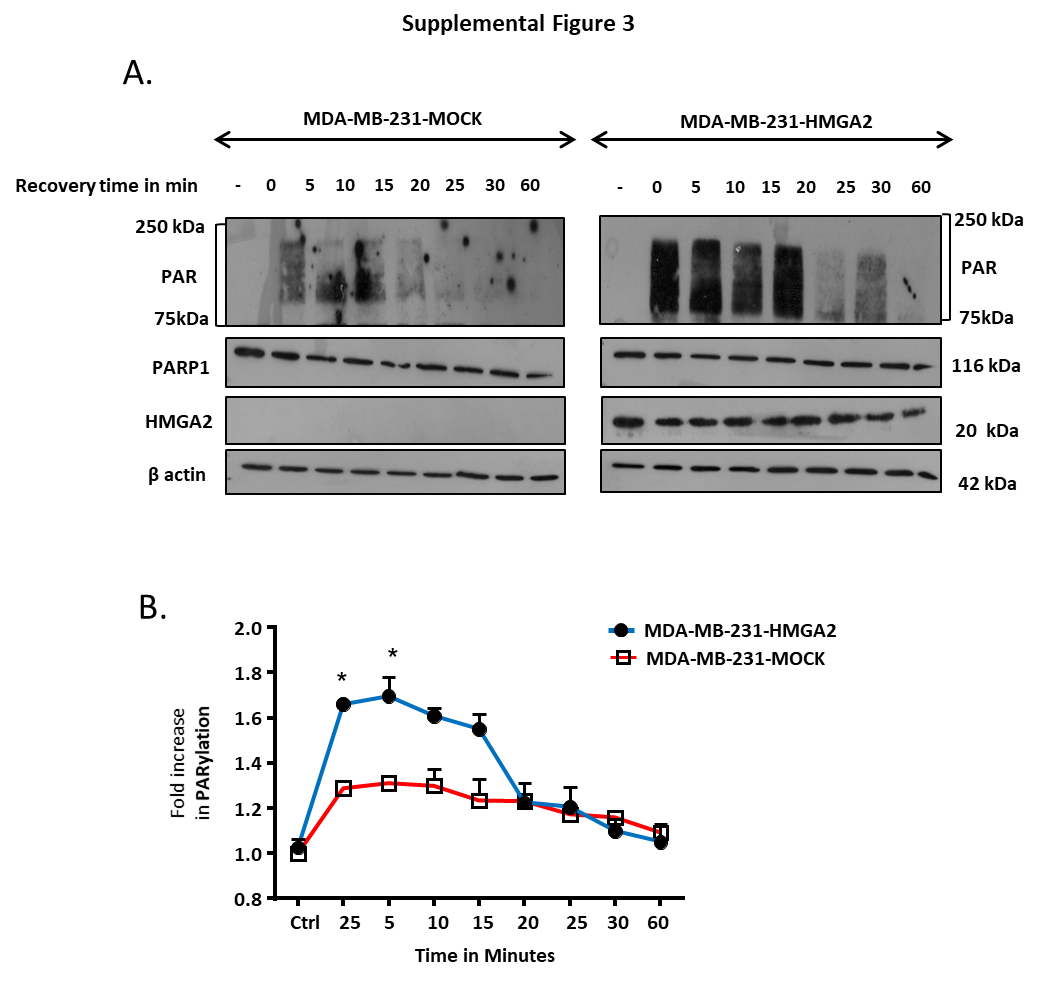


**Suppl. Fig. 3: MMS-induced cellular PAR levels normalize after 30 min recovery time. A.** Representative Western blots are shown for PAR detection following MMS treatment. Normal medium recovery time kinetics for 0, 5, 10, 15, 20, 25, 30 and 60 min after MMS treatment (30 min, 4 mM) of MDA-MB-231-HMGA2 and MDA-MB-231-Mock cells demonstrated that PAR levels were drastically reduced after 30 min, independent of cellular HMGA2 levels. **B**. PAR detection was quantified by densitometry, normalized to corresponding actin controls and presented as PARylation in the graph. Data are shown as mean +/- SEM; *p<0.05.


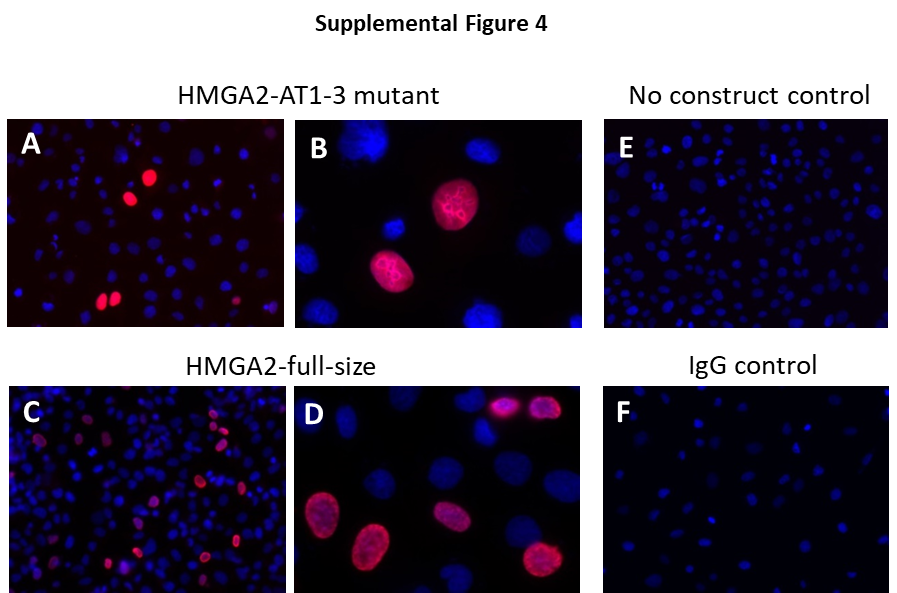


**Suppl. Fig. 4: Flag-immunofluorescence on C1 cells transiently transfected with the AT1-3 mutant (A, B) or full-size (C, D) HMGA2 constructs.** Representative IF images are shown. These constructs were used for the quantification of DNA damage-induced PARylation. All constructs resulted in nuclear protein expression as determined here with anti-Flag antibody. Non-transfected control cells (**E**) as well as transfected cells probed with the isotype control antibody (**F**) did not show any fluorescence. Magnification: 100 x for A, C, E, F and 200 x for B,D.


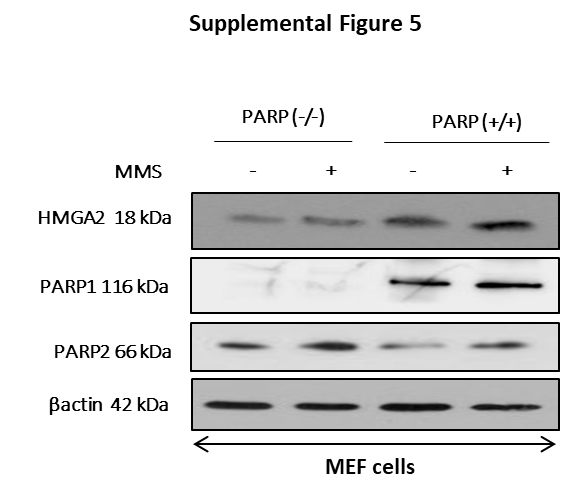


**Suppl. Fig. 5: PARP1 knock-out MEF cells (PARP1 -/-) lack PARP1 protein but express PARP2 and HMGA2 proteins.** Western blot confirming the lack of PARP1 protein expression in PARP1 knock-out (KO) MEF cells (PARP1-/-). PARP2 protein and HMGA2 protein were consistently expressed in both, PARP1 KO and PARP1 wildtype MEF cells.


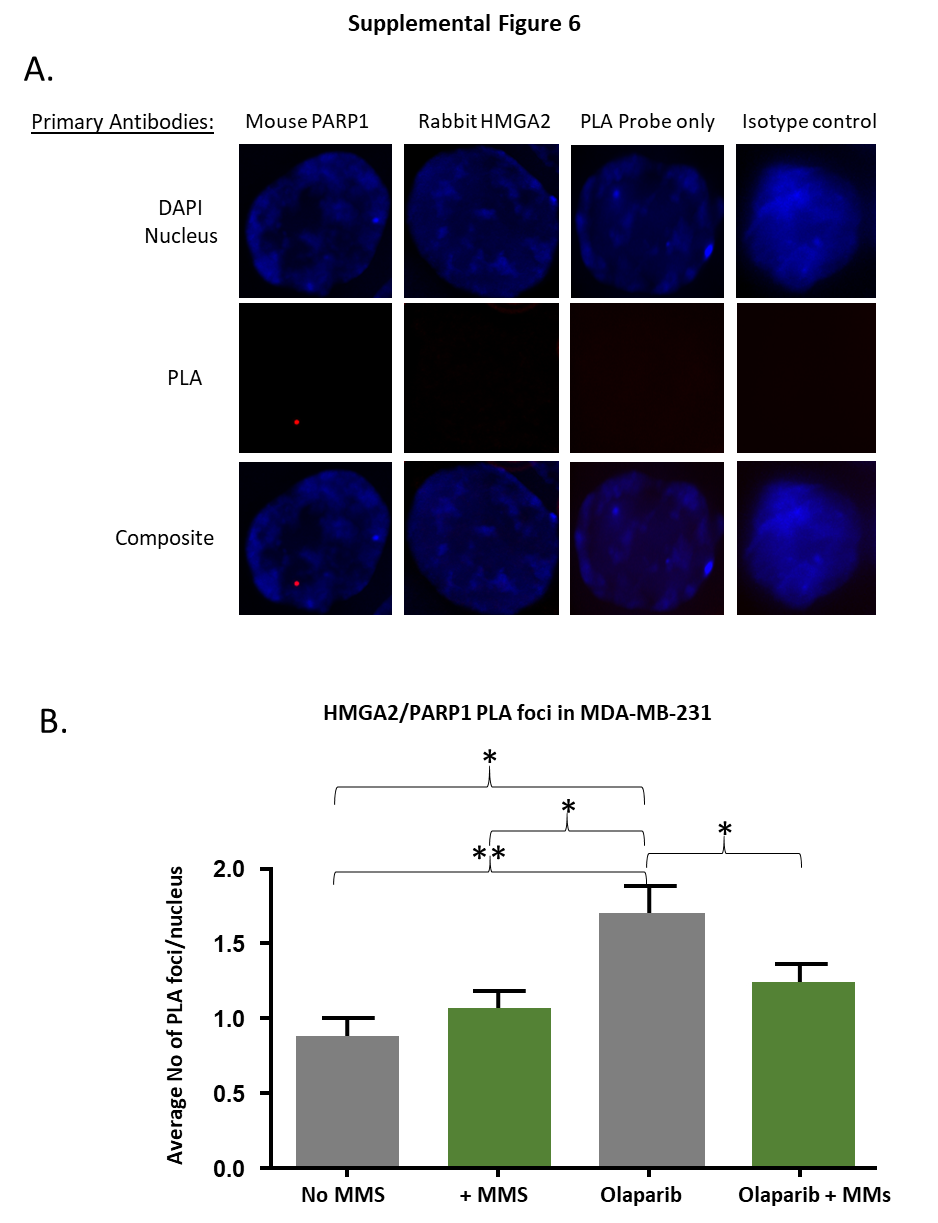


**Suppl. Fig. 6: Proximity ligation assays (PLA) demonstrate HMGA2/PARP1 colocalization in MDA-MB-231 cells. A.** Representative PLA controls for the Proximity Ligation Assays in Fig.4C. Single primary antibodies for PARP1 and HMGA2 as well as the isotype control antibodies (shown here for the Mouse IgG) and the PLA probe alone were used as negative controls for the PLA assay. The average number of background signals from these controls were presented in the graph in Fig. 4D, together with the PLA foci representing protein proximity for PARP1 and HMGA2. **B.** PLA assay for HMGA2 and PARP1 was performed on MDA-MB-231 breast cancer cells. The average number of PLA foci was quantified from 50 nuclei/treatment group. Data are shown as mean +/- SEM; **p<0.01, *p<0.05.


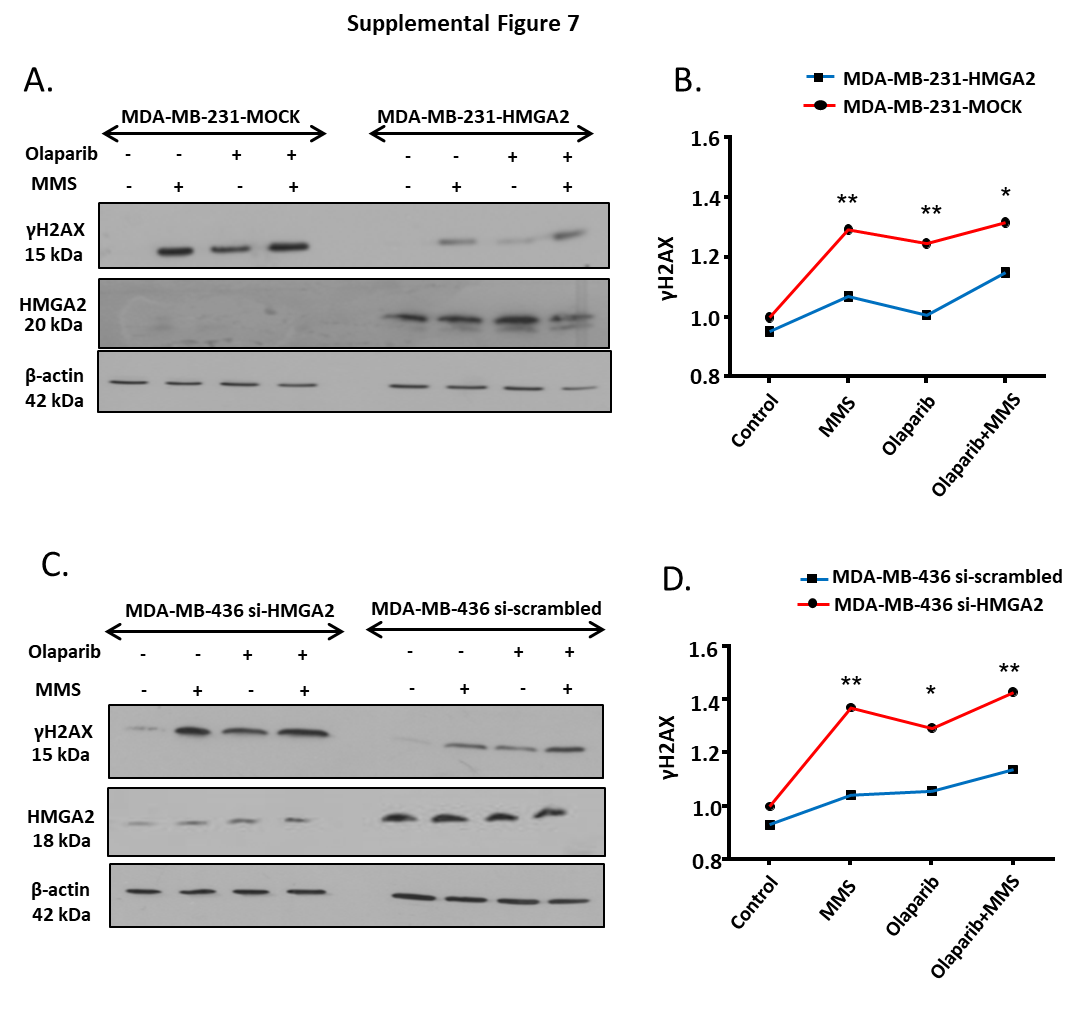


**Suppl. Figure 7: HMGA2 knockdown along with inhibition of PARP-1 activity increases DNA double strand breaks (γH2AX). (A, C)** MDA-MB-231 (HMGA2 overexpressing and Mock clones) and MDA-MB-436 (siHMGA2 treated or non-silencing treated) cells were treated with Olaparib for 24 h and exposed to low MMS concentrations of 0.3mM for 24 h before proteins were isolated. Low cellular HMGA2 expression conferred higher γH2AX levels upon DNA damage induction by MMS. **(B, D)** The values obtained by densitometry analysis were normalized to corresponding controls and were presented in the graph as γH2AX compared to MMS untreated cells. Data were shown as SEM +/- from three independent experiments and p values were determined using Anova analysis; *p<0.05 was considered significant. ** p<0.01.


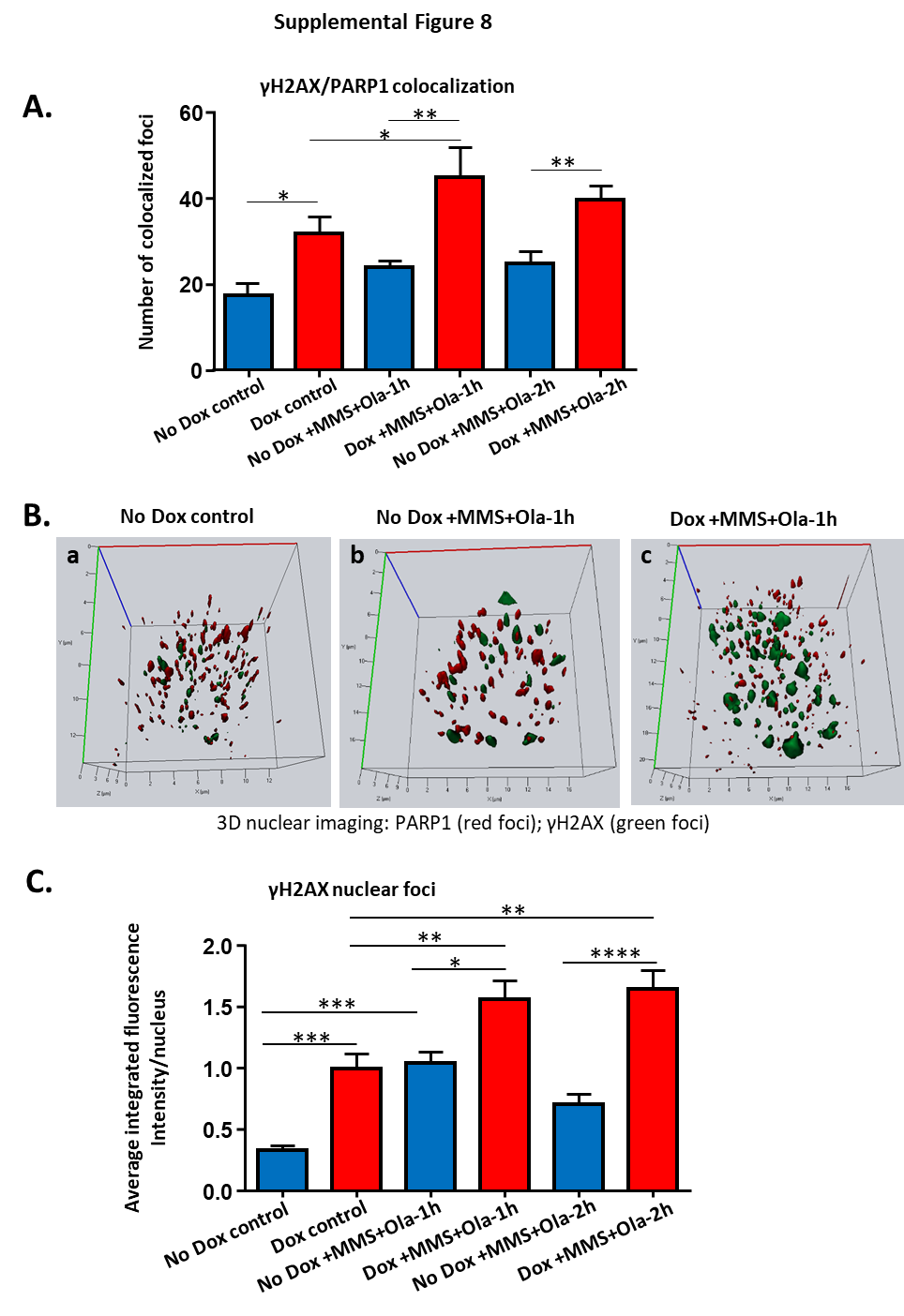


**Suppl. Figure 8: HMGA2 knockdown increases PARP1 co-localization with the DNA damage marker γH2AX.** Following 5 days of Dox-induced shRNA-mediated HMGA2 knockdown, C1 cells were treated with Olaparib for 4 h prior to exposure to 4mM MMS for 20 min. After removal of MMS, cells were recovered in fresh medium for 1 h and 2 h, then fixed in paraformaldehyde and co-immunofluorescence was performed for PARP1 (red) and γH2AX (green). **(A)** Following 3D nuclear imaging, Image J software was used to quantify co-localization for PARP1 (red) and γH2AX (green) in the different treatment groups. Knockdown of HMGA2 (grey columns) resulted in increased PARP1/γH2AX co-localizing foci. The association of PARP1 with DSB sites further increased upon MMS and Olaparib treatment. **(B)** Representative examples for 3D nuclear images are shown with a no treatment control as reference (*a*). Cells fixed after 1 h recovery from treatment with MMS and Olaparib reveal DSB sites with higher fluorescence intensity (bigger green foci) in Dox-treated cells (*c*) compared to cells with endogenous HMGA2 levels (*b*). **(C)** To assess if the increased co-localization of PARP1 with γH2AX foci upon combined MMS/ Olaparib treatment in HMGA2 depleted cells (shown in A.) is associated with increased DSBs we quantified the average integrated density of nuclear γH2AX foci at 1 h and 2 h recovery. The integrated optical density takes into account the combination of the number and size of foci to quantify fluorescence [Böcker W and Iliakis G. Computational Methods for Analysis of Foci: Validation for Radiation-Induced γ-H2AX Foci in Human Cells. Radiation Research, Jan 2006 : Vol. 165, Issue 1, pg(s) 113- 124]. In cells depleted of HMGA2 (+Dox), we detected a significantly higher average integrated density of nuclear γH2AX foci which remained elevated during recovery. This indicated that increased PARP1-DNA association under Olaparib/ MMS co-treatment leads to increased DSBs. Data are shown as mean +/- SEM; ***p<0.0001, ***p<0.001, **p<0.01, *p<0.05.


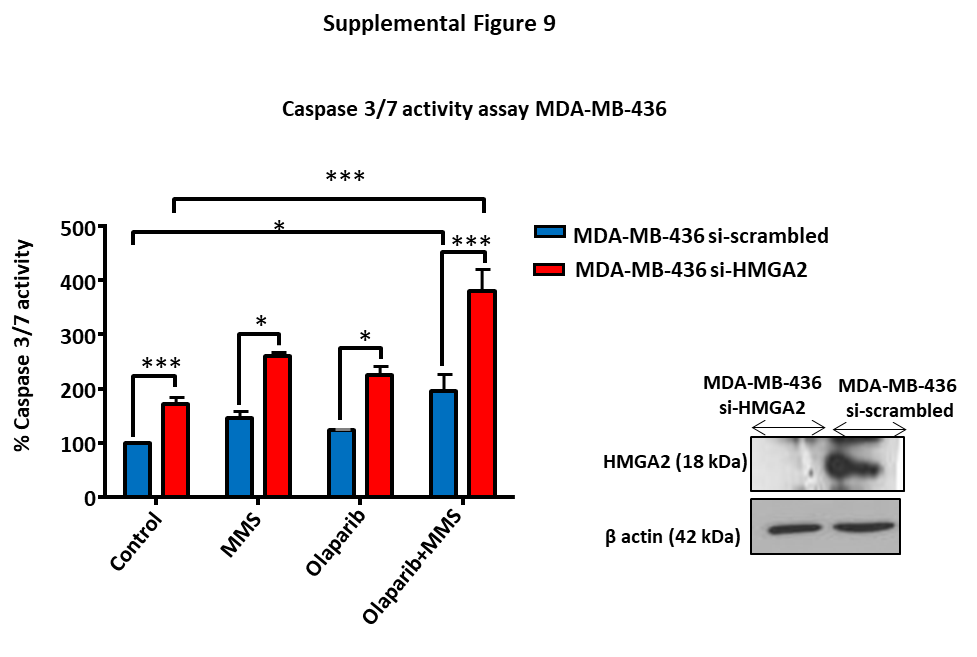


**Suppl. Figure 9: HMGA2 silencing increases apoptosis in MDA-MB-436 cells.** MDA-MB-436 cells were treated with Olaparib (20 µM) for 24 h prior to MMS treatment (4 mM, 30 min) with and without silencing of endogenous HMGA2 by siRNA and subjected to caspase 3/7 apoptosis assays. HMGA2 silencing increased caspase 3/7 activity and this effect was strongest after combined MMS and Olaparib treatment. The image on the right shows a representative Western blot for siRNA-mediated HMGA2 KD in MDA-MB-436 cells. The values obtained by colorimetric quantification in the untreated controls were set to 100%. Data are presented as % caspase 3/7 activity compared to MMS untreated cells. Data were shown as mean SEM +/- from three independent experiments. *p<0.05; ***p<0.001.
